# The power of data-driven ASSISTance in personalized testing for coronary artery disease

**DOI:** 10.1093/ehjdh/ztae057

**Published:** 2024-07-30

**Authors:** Ali Wahab, Ramesh Nadarajah

**Affiliations:** Leeds Institute of Data Analytics, University of Leeds, 6 Clarendon Way, Leeds, LS2 9DA, UK; Leeds Institute for Cardiovascular and Metabolic Medicine, University of Leeds, 6 Clarendon Way, Leeds, LS2 9DA, UK; Department of Cardiology, Leeds General Infirmary, Leeds Teaching Hospital NHS Trust, Great George Street, Leeds, LS1 3EX, UK; Leeds Institute of Data Analytics, University of Leeds, 6 Clarendon Way, Leeds, LS2 9DA, UK; Leeds Institute for Cardiovascular and Metabolic Medicine, University of Leeds, 6 Clarendon Way, Leeds, LS2 9DA, UK; Department of Cardiology, Leeds General Infirmary, Leeds Teaching Hospital NHS Trust, Great George Street, Leeds, LS1 3EX, UK

## Introduction

Nearly 200 million people globally suffer from coronary artery disease (CAD),^1^ half of whom initially present with chest pain.^1,2^ Current guidelines emphasize the use of non-invasive investigation (Class 1 recommendation, Level of Evidence B) for suspected CAD.^3,4^ Consequently, the use of functional stress tests and anatomical imaging through coronary computing tomography angiography (CTCA) for patients at low to intermediate risk of CAD has increased.^3,5^ It is now recognized that, as well as providing diagnostic information, these imaging modalities can improve outcomes by enabling the implementation of preventative therapies.^6^ Whether to decide between a functional and anatomical test is a complex process incorporating multiple variables, and prone to the biases of an individual clinician. Such biases may lead to decision-making that leads to poorer outcomes for patients or disparities between patient groups.^7^ Data-driven machine learning may be able to identify where testing strategies differ from the optimal approach, thus reducing disparities, and achieving improvement in long-term cardiovascular outcomes.

## Discussion

The linked study published by Oikonomou and colleagues in this issue of the journal explores cardiovascular outcomes of stable chest pain patients when comparing testing strategies employed in real-world care with the strategies recommended by the Application of Anatomical vs. Stress teSting decision Support Tool (ASSIST).^8^ The authors previously developed ASSIST to predict personalized benefit of anatomical vs. functional testing through phenomapping and an extreme gradient boosting algorithm,^9^ using participant level data from the PROMISE (Prospective Multicentre Imaging Study for Evaluation of Chest Pain) trial, with external validation in the SCOT-HEART (Scottish Computed Tomography of the Heart) trial.^6,10^ In trial populations, the testing strategy recommended by ASSIST was associated with improved outcomes, but this study seeks to address whether this is robust in real-world practice, where decisions are not made as a consequence of random allocation.

From 134 216 individuals in Yale health system [anatomical *n* = 4030 (3%), functional *n* = 130 196 (97%)] and 3901 individuals in UK Biobank [anatomical *n* = 581 (14.9%), functional *n* = 3320 (85.1%)], 11 391 (8.5%) and 484 (12.3%) experienced the primary composite outcome all-cause death and acute myocardial infarction over a median follow-up of 4.9 years and 5.4 years, respectively. ASSIST would have projected better outcomes from anatomical testing for 18% of individuals in the Yale health system and 21.2% of the UK Biobank cohort, thus in both cases in excess of real-world practice, with female sex and a history of diabetes significantly associated with a lower likelihood of undergoing ASSIST-recommended testing strategy. Patients who underwent testing concordant with ASSIST recommendation were less likely to experience the primary outcome [Yale health system: adjusted HR 0.81 (95% CI 0.77–0.85); UK Biobank: 0.74 (95% CI 0.60–0.90)] after propensity score adjustment including risk factors. But why should a difference in testing strategy result in better outcomes? In a mechanistic sub-study, the authors leverage PROMISE data to demonstrate that ASSIST-concordant testing with CTCA was associated with a greater frequency of identifying high risk anatomic disease, thus potentially enabling better medical prevention strategies.

**Figure 1:**
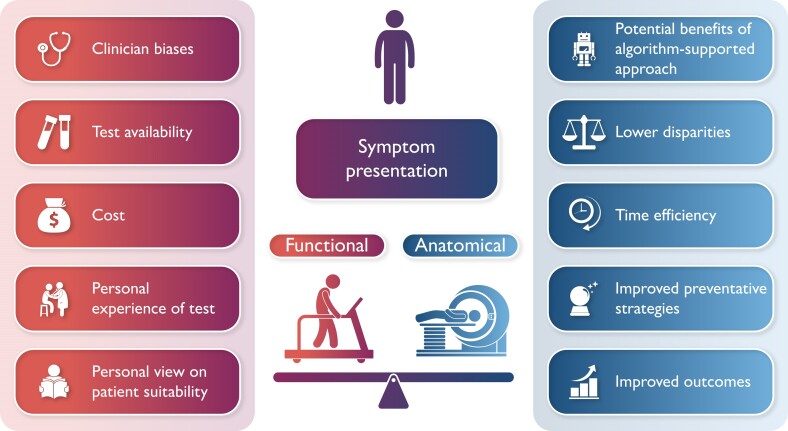
Factors influencing selection of anatomical vs functional testing in chest pain evaluation.

There are limitations to the study. This real-world analysis was heavily skewed towards functional testing. That the dataset is skewed to such a great extent implies that factors beyond those that can be accounted for likely influenced clinical decision-making. The use of all-cause mortality in the primary endpoint (a decision based on the lack of granularity in the Yale health system data) means that some of the benefit observed from an ASSIST-guided strategy may not be mechanistically linked to testing strategy. Furthermore, the exact role of ASSIST in the clinical pathway is still to be determined. ASSIST allows the projection of a new participant to a simulated PROMISE trial setting to predict the strategy that would work best in that setting, and its role could range from quantifying human-led diversion from an algorithmic approach to test selection to informing clinician decision-making between the different testing strategies (*Figure 1*).

With multiple challenges and disparities present in investigation of suspected CAD,^11^ the use of data-driven support tools has the potential to improve the fairness of care and patient outcomes. The authors should be congratulated for using real-world data to identify disparities in testing strategies that may disadvantage certain individuals, and thus the value of incorporating (objective) data-driven methods to inform decision-making. These findings add weight to the notion that we now need a prospective evaluation of a data-driven-informed selection of testing strategy in patients presenting with stable chest pain.
